# Case report and review of literature: IgG4-gastroduodenitis in upper GI Crohn’s disease: two separate entities or just a marker of disease severity?

**DOI:** 10.3389/fmed.2024.1388940

**Published:** 2024-07-19

**Authors:** Valérie Desmedt, Jeroen Geldof, Anne Hoorens, Triana Lobaton

**Affiliations:** ^1^Department of Gastroenterology and Hepatology, University Hospital Ghent, Ghent, Belgium; ^2^Department of Pathology, University Hospital Ghent, Ghent, Belgium; ^3^Department of Internal Medicine and Pediatrics, Ghent University, Ghent, Belgium

**Keywords:** granulomatous disease, IgG4-related disease, Crohn’s disease, gastritis, anti-TNF, upper GI tract

## Abstract

A 20-year-old man was presented with ulcerative gastritis and duodenitis complicated by pyloric stenosis. *Helicobacter pylori* infection was excluded, and the lesions did not respond to treatment with proton pump inhibitors. No other parts of the intestinal tract showed signs of inflammation. Histopathological review showed signs of chronic inflammation with granuloma formation. A tentative diagnosis of isolated upper gastrointestinal (UGI) Crohn’s disease was performed. However, additional work-up revealed significantly positive IgG4 staining as well as elevated IgG4 serum levels. Since granulomatous disease is unlikely in IgG4-related disease, an eventual diagnosis of overlapping IgG4-related disease and Crohn’s disease (CD) was performed. Treatment with systemic steroids and anti-TNF in combination with azathioprine led to rapid symptomatic improvement. In this article, we review the available literature on IgG4-related gastroduodenitis, granulomatous gastritis, and upper GI CD. We suggest the possibility that IgG4-infiltration may be a marker of severely active inflammatory bowel disease rather than a separate disease entity.

## Introduction

Gastroduodenal inflammation is a common finding when performing esophagogastroduodenoscopy (EGD). In many patients, an obvious cause, such as *Helicobacter pylori* (HP) or non-steroidal anti-inflammatory drug (NSAID)-associated peptic ulcer disease, can be identified (1). First-line treatment consists of proton pump inhibitors (PPIs) or HP eradication (2). However, some patients do not respond, and a broad range of more rare disease entities need to be explored. We report a case of treatment-refractory ulcerative gastritis and duodenitis complicated by pyloric stenosis, in which we evaluated a broad differential diagnosis based on the clinical course and pathological findings.

## Case report

A 20-year-old man with a previously unremarkable medical history presented to the outpatient clinic with epigastric pain and 10 kg weight loss over the past 6 months. He also reported early satiety during meals, nausea without vomiting, and looser stools for a couple of months. The patient was an active smoker and had regular alcohol consumption, but denied illegal substance use. He worked as a logistic assistant in the shipping industry. He did not recently use NSAIDs or any maintenance medical treatment. Familial history included colorectal cancer (paternal grandfather, at the age of 60) and a perianal fistula (father). Vital parameters were normal, and physical examination showed no abdominal abnormalities. No pathological lymph nodes were palpable.

Initial laboratory testing showed no anemia. White blood cell count was within normal range and there was mild thrombocytosis (394,000 10E3/μL). The serum electrolytes were within normal limits, except for mild hypomagnesemia (0.61 mmol/L, 0.70–1.05 mmol/L). There was mild elevation of aspartate aminotransferase (AST) (84 U/L; reference <37 U/L) and alanine aminotransferase (ALT) (92 U/L; reference <40 U/L) with no alterations in other liver function tests. IgE titer was remarkably elevated (758 kU/L; reference 0–100 kU/L). Fecal calprotectin was slightly elevated (130.9 mg/kg, reference <50 mg/kg).

A few months earlier, the patient had already undergone an EGD for his complaints at a different center. This revealed a bumpy and erosive appearance of the gastric mucosa and bulboduodenal ulcerations with stenosing effect, causing gastric outlet subobstruction. The biopsies showed chronic active, HP-negative gastritis and bulbitis, in the absence of PPI intake. There were no signs of malignancy, and no granulomas were observed. Pantoprazole 40 mg BID was started.

Control EGD after presentation at our center showed similar macroscopic findings despite PPI treatment: diffuse gastritis with gastric outlet stenosis due to large ulcers at the transition between pylorus and bulbus, which could only be passed with a nasogastric endoscope (diameter 5.4 millimeter) (Figure 1). Repeated extensive biopsy sampling confirmed persistent acute bulbitis and HP-negative, chronic active gastritis. Periodic acid-Schiff staining gave no arguments for Whipple’s disease.

**Figure 1:**
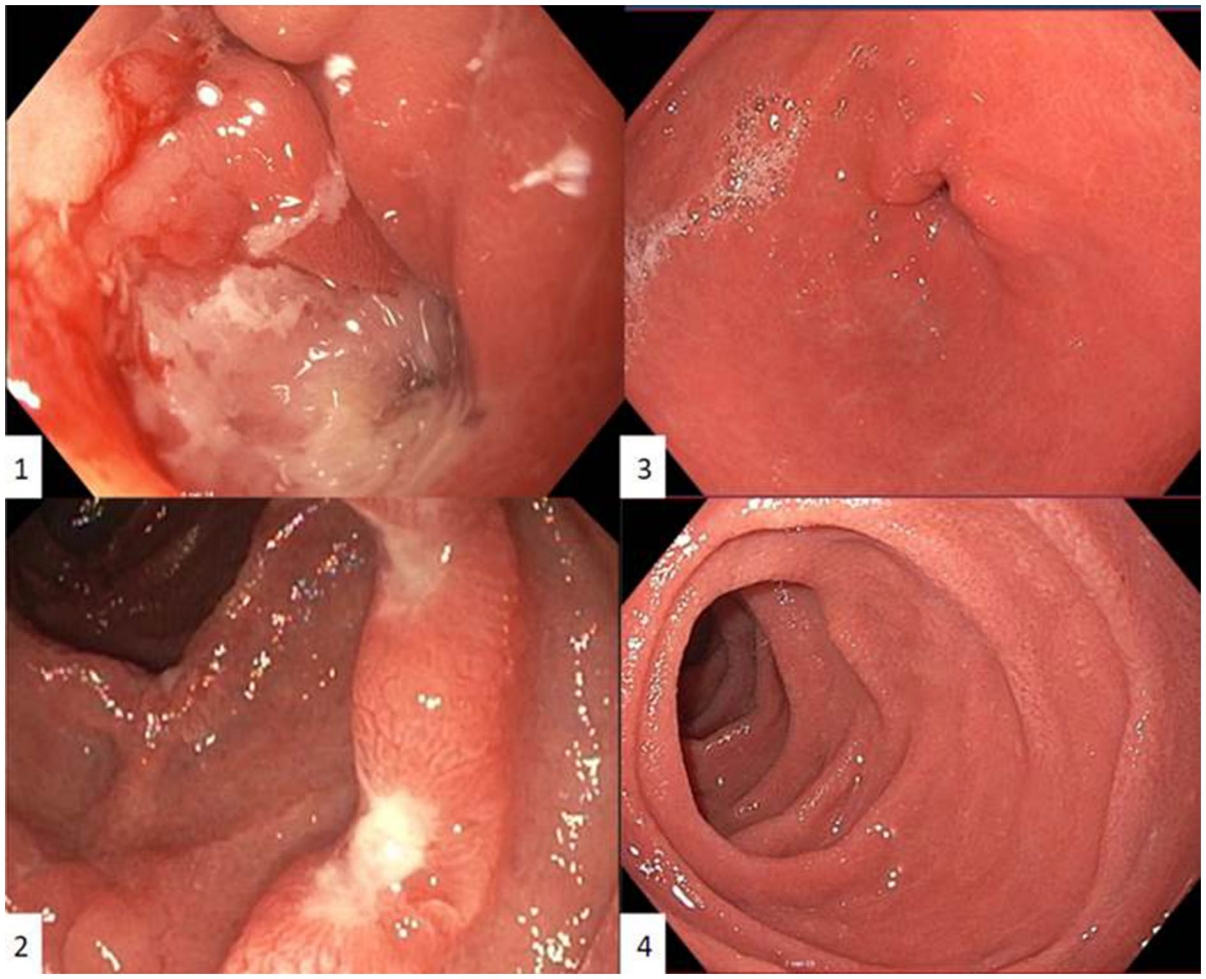
First esophagogastroduodenoscopy in our centre showing an ulcerative gastritis with gastric outlet stenosis due to large ulcers at the transition between pylorus and bulbus, which could only be passed with a nasogastric endoscope (diameter 5.4 millimeter) **(1)** and duodenal ulcerations with intermediate normal appearing mucosa **(2)**. Control esophagogastroduodenoscopy six months after induction with Infliximab and azathioprine showing no residual inflammation in the antrum **(3)** or duodenum **(4)**. Pyloric opening and transition between pylorus and bulbus could be easily passed with a gastric endoscope (diameter 9.9 millimeter).

An abdominal computed tomography (CT), followed by additional investigations including ileocolonoscopy with ileal and colonic biopsies and a magnetic resonance (MR) enterography, did not show other locations of intestinal inflammation. An additional video capsule endoscopy to screen for more distal small bowel inflammation could not be performed because the capsule could not be advanced beyond the pyloric stenosis despite endoscopic maneuvers.

Screening for Zollinger–Ellison (ZE) syndrome showed a mildly elevated serum gastrin level (339 ng/L) on PPI and 68Ga-DOTA-1-NaI3-octreotide (DOTANOC) positron emission tomography (PET)-CT showed no elevated somatostatin receptor expression. Endoscopic ultrasound (EUS) of the pancreas and MR enterography showed no arguments for a primary neuro-endocrine tumor. There were no histopathological arguments for autoimmune gastritis, and anti-intrinsic factor antibodies and anti-parietal cell antibodies were negative. Intestinal tuberculosis was excluded by a negative interferon-gamma release assay (i.e., QuantiFERON-TB) and normal X-ray of the thorax. Serum calcium and serum angiotensin-converting enzyme (ACE) levels were within normal limits, which made sarcoidosis less likely.

After this profound work-up, the patient was referred to the IBD unit of our hospital for further investigation. In order to establish a diagnosis, a repeated EGD with biopsies showed erosions in the stomach with the known stenosis of the pylorus, a large ulcer at the transition from the pylorus to the bulbus and multiple punched-out ulcerations in the duodenum. The gastric biopsies now showed a few small non-caseating granulomas, suggestive of Crohn’s disease (CD), since other causes of granulomatous gastritis (GG), i.e., sarcoidosis, malignancy, and infectious diseases such as tuberculosis or Whipple’s disease, had already been thoroughly ruled out (Figure 1). In addition, because of persistent ulcerative gastritis with stenosis, despite high doses of PPI, immunoglobulin G4 (IgG4) staining was performed to rule out IgG4-related disease, and the number of IgG4-positive plasmocytes was found to be significantly elevated on both gastric and duodenal biopsies. In the corpus of the stomach, the number of IgG4-positive plasma cells was highest, with more than 50 IgG4-positive plasma cells per high power field (HPF) and an IgG4/IgG ratio above 40%. An elevated IgG4 was seen in serum (194 mg/dL, reference values 8–140 mg/dL). Based on these clinical, serological, radiological, and histopathological findings, the presumptive diagnosis of an overlap of gastroduodenal CD and IgG4 disease was performed, as granulomas are unlikely in IgG4-related disease. Initially, systemic steroids were refused by the patient. Budesonide (9 milligram/day) was started without any effect on the complaints. Consequentially, a methylprednisolone 32 mg/day tapering course was started, and because of the suspected upper gastrointestinal (UGI) CD, anti-tumor-necrosis factor alpha (anti-TNFα) treatment with adalimumab was initiated early in the disease course. This approach led to rapid symptomatic improvement.

**Figure 1:**
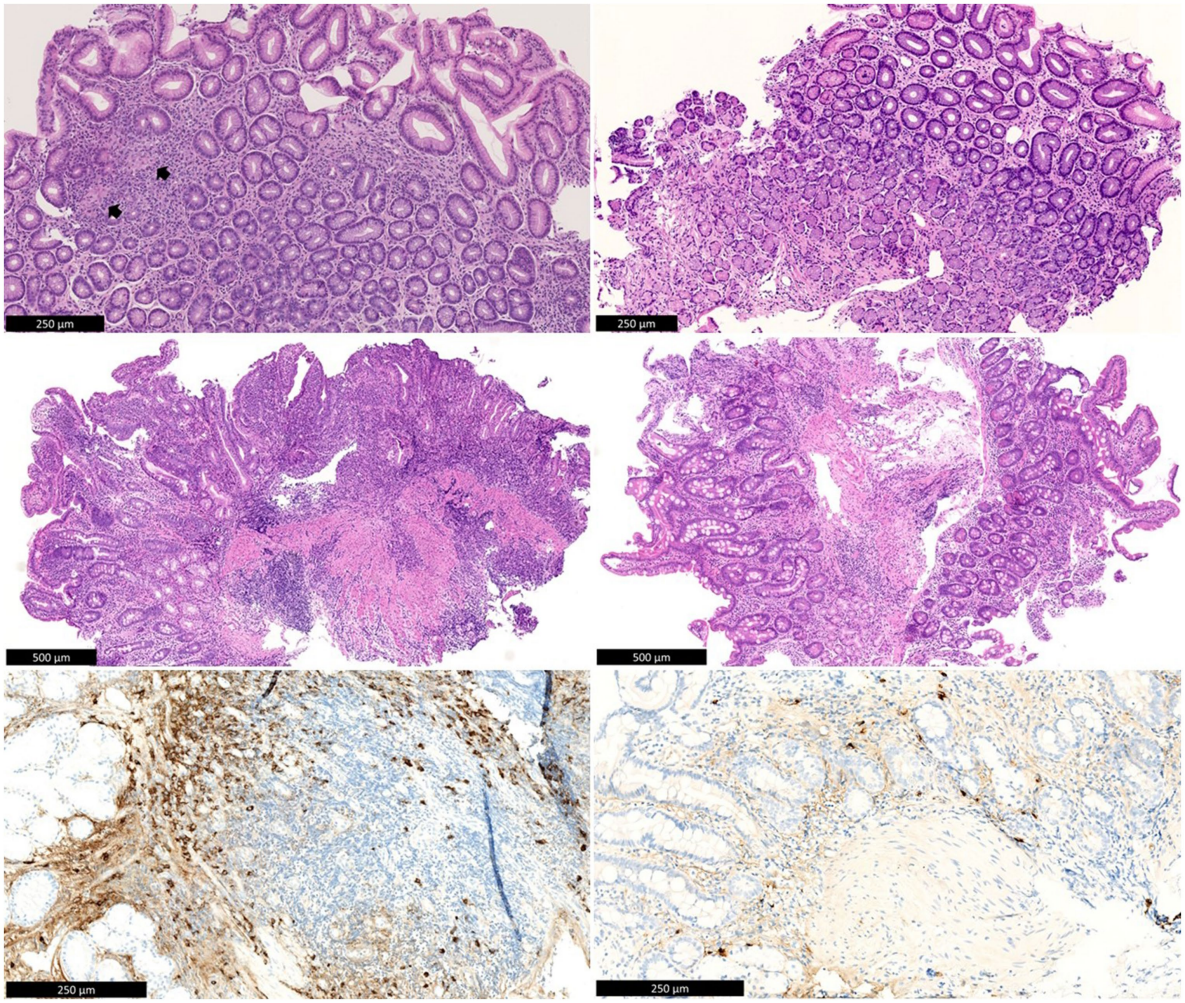
Top left: Antral mucosa with chronic inactive gastritis and two non-necrotic granulomas (arrows). Middle left: Duodenal bulb mucosa with active chronic inflammation with erosion. Below left: IgG4 immunohistochemistry shows numerous IgG4-positive plasma cells in the duodenal mucosa (>100/HPF). There is background staining in the stroma, as is often seen with high serum IgG4 levels. Top right: Antral mucosa after treatment, without significant inflammation. Middle right: Duodenal mucosa after treatment, without significant inflammation. Below left: IgG4 immunohistochemistry shows rare residual IgG4-positive plasma cells in the duodenal mucosa. There is also much weaker background staining in the stroma compared to before treatment.

A control EGD (performed when the patient was still under CS) confirmed a clear endoscopic response with resolution of large ulcerations but still persistent diffuse gastroduodenitis with erosions. Because of the positive IgG4 staining, after a review of the literature, it was decided to switch adalimumab to infliximab in combination with azathioprine. After induction with Infliximab, EGD now showed an increased response, with only some residual erythema in the antrum and now a normal pyloric opening that could be passed with a gastric endoscope (diameter 9.9 millimeter). No abnormalities were visualized in the duodenal bulbus. However, active chronic inflammation with increased IgG4-positive plasma cells (>100 IgG4-positive plasma cells/HPF) remained present in the duodenal biopsies. Gastric biopsies showed inactive chronic gastritis with up to 14 IgG4-positive plasma cells/HPF. Control EGD 6 months after induction showed no macroscopic abnormalities, with only mild chronic and inactive antritis in the biopsies without granulomas (Picture 1). IgG4-plasmocyte count was also normalized (only 7/HPF in the duodenum and 2/HPF in the gastric biopsies).

## Review of literature

In this case report, we suggest an overlap between gastric CD and IgG4-related disease (IgG4-RD). A review of literature was performed below to summarize the diagnosis and treatment of both entities.

### Granulomatous gastritis

GG is rare, with a prevalence between 0.08 and 0.35%. Histopathological evaluation reveals non-necrotizing epithelioid cell granulomas. GG is commonly classified as an uncommon inflammation pattern and not as a distinct diagnosis. Therefore, it is unclear whether GG should be called idiopathic or if extensive work-up is needed to rule out underlying causes (3). For example, GG has been linked to CD, sarcoidosis, foreign bodies, neoplasms, and vasculitis. Furthermore, multiple infectious diseases are demonstrated in GG, i.e., aspergillosis, tuberculosis, parasites, histoplasmosis, and Whipple disease. CD, sarcoidosis, and tuberculosis are most prevalent (4).

Liang et al. investigated more than 142,000 gastric biopsies. GG was confirmed in 0.19%. There was no correlation between HP and GG, with a significantly higher amount of HP in the non-GG biopsies (*p* < 0.001) (4). On the contrary, other studies suggest HP infection as a possible causal factor for GG pathogenesis, in which eradication can lead to granuloma resolution (5, 6). Liang et al. (4) identified an isolated GG in 32% of the cases, of which 59.3% eventually were diagnosed with CD. GG-associated CD was more prevalent in male and young patients. Furthermore, single, small, or antral granulomas are more likely in CD (4). Shapiro et al. (3) suggest also taking into account the background inflammatory pattern of the gastric mucosa to help categorize GG.

### Upper gastrointestinal (UGI) Crohn’s disease

CD is a chronic inflammatory bowel disease characterized by a segmental and transmural involvement of the bowel wall that can affect any part of the intestinal tract. Crohn’s gastritis is commonly associated with duodenitis, is referred to as “gastroduodenal CD,” and is defined according to Montreal’s Classification as L4 (7). Gastroduodenal CD is reported in 0.5 to 4.0% of all CD cases (8–11). Isolated gastric involvement is rare, affecting less than 0.07% of all CD cases (12). Almost 60% of patients with gastroduodenal CD have had previous inflammation elsewhere in the GI tract, and one-third of patients with isolated upper GI CD (UGI-CD) at diagnosis will develop distal disease later in life (8, 10, 13). UGI-CD is most frequently diagnosed in the fourth decade of life, although some reports show that the disease is more common in children than in adults (8, 9, 13). A cross-sectional study has demonstrated that proximal CD affects younger and non-smoking patients. In these patients, concomitant ileal disease and stenosing behavior are more present and are associated with a higher probability of undergoing abdominal surgery. Upper GI CD can also be associated with colonic inflammation, although less frequently (14).

#### Diagnosis

UGI-CD is diagnosed based on a combination of clinical presentation, biochemical signs of inflammation, and endoscopic evaluation with biopsy and histopathological evaluation (12, 15). Nugent and Roy grouped these investigations and formulated diagnostic criteria for gastroduodenal CD: (1) demonstration of non-caseating granulomatous inflammation in the stomach or duodenum in the absence of other systemic granulomatous disorders, with or without more distal intestinal inflammation; (2) endoscopic or radiographic findings of diffuse inflammation in the stomach or duodenum consistent with CD in a patient with confirmed CD of the GI tract (11).

Endoscopic evaluation is indicated in every CD patient with upper GI symptoms according to international guidelines by the European Crohn’s and Colitis Organization (ECCO) and the European Society of Gastrointestinal and Abdominal Radiology (ESGAR) (16). However, due to the potential asymptomatic presentation, upper GI disease might be missed without routine endoscopic evaluation. For example, Annunziata et al. reported upper CD involvement in 16% of the patients, of whom 63% were asymptomatic (17). Routine EGD identifies mild macroscopic inflammation in 30 to 64% of CD patients and microscopic inflammation in up to 70% of patients (9).

UGI-CD is most frequently localized at the antrum and proximal duodenum. The proximal stomach is usually not affected (10, 18). Endoscopic lesions are not specific and are similar to CD lesions in the more distal parts of the GI tract. These include mucosal edema, longitudinal or irregular erosions, and ulcers that may be deep and punched out. Also, nodularity of the mucosa, cobblestone or bamboo-joint-like appearance, stenoses and fistulae are described (10, 16, 19).

Histologic features of CD include patchy or focal chronic inflammation in combination with focal crypt architectural abnormalities, preservation of mucin at active sites and the presence of non-caseating granulomas (20). None of these findings are pathognomonic, and biopsy findings are often non-specific in upper GI CD (8). The main histological findings described in the CD of the stomach and duodenum are acute and chronic inflammation, focal inflammatory changes, lymphoid aggregates, mucosal-muscular fibrosis, chronic HP-negative gastritis, focal gastritis, epithelioid granulomas, and duodenitis with or without granulomas. Because of the focal/patchy distribution of the disease, biopsies should be taken from macroscopically normal areas as well as affected areas in the stomach. Prevalence of non-caseous granuloma varies according to different publications, and percentages between 0 and 83% of the cases are reported. The finding of granuloma is not a definitive criterion of CD (8, 19, 21). Exclusion of other causes of granulomatous lesions is therefore necessary (8, 16).

The main differential diagnoses of UGI-CD are peptic ulcer disease, gastrinoma, ZE syndrome, Ménétrier disease, lymphoma, tuberculosis, sarcoidosis, gastric syphilis, plastic lymphoma, amyloidosis, and collagen diseases (8, 22). Intestinal tuberculosis should be ruled out, particularly in endemic regions. Gastric and duodenal tuberculosis are rare (0.4–2% and 2–2.5%, respectively) and are usually associated with pulmonary tuberculosis or immunodeficiencies (10). In Ménétrier’s disease, the entire stomach can be involved and ulcerations do occur. However, Ménétrier disease does not cause transmural damage. Last but not least, malignant and infiltrative processes need to be ruled out by the histological findings (8).

#### Treatment

The first line of treatment consists of PPIs with or without steroids (8). PPIs are only a complementary treatment as they do not affect chronic inflammation (10). Further, the medical treatment for UGI-CD does not differ substantially from the rest of the CD locations. Thiopurines can be used as a maintenance treatment (8).

Additionally, in the era of biological treatment, treatment with anti-TNF has shown good results. In the ACCENT I trial, 56% of patients with gastroduodenal CD responded to infliximab within 2 weeks of therapy, similar to the response observed in other locations of intestinal CD inflammation (23). Adalimumab is a valid treatment option as well, with a satisfactory response, according to several case reports (17, 24).

Short, secondary, gastroduodenal strictures can be treated with endoscopic balloon dilation, intestinal resection, or strictureplasty on a case-to-case basis (25). Gastric outlet obstruction, fistula, upper GI hemorrhage, and abscess formation are indications for surgical treatment (8). The most common indication for surgery is small bowel obstruction, requiring surgery in up to 91% of patients. Options for surgical management of complicated duodenal CD include bypass, stricturoplasty, or resection. However, resectional surgery has become exceptional due to excessive morbidity (26, 27).

### IgG4-related upper GI disease

IgG4-RD was described for the first time in the early 2000s and is characterized by fibrotic lesions in multiple organs such as salivary glands, lacrimal glands, pancreas, or retroperitoneum (28, 29). It can mimic many inflammatory, infectious, and malignant diseases (30). Upper GI IgG4-RD is rare, but exact numbers of prevalence are lacking. It is unclear whether the risk of gastric malignancy is increased in gastric IgG4-RD. In a Japanese observational study, two out of eight patients with gastric IgG4-RD had a concurrent malignancy, but the study size was too small to conclude (31).

#### Diagnosis

Diagnosing upper GI IgG4-RD is difficult, as it can mimic peptic ulcers, gastrointestinal stromal tumors (GISTs), submucosal tumors, and malignancy. Recently, even a case report of gastric IgG4-RD presenting as a collagenous gastritis was published (32). This diagnostic difficulty may delay adequate treatment and pose a risk of unnecessary treatment and even resection. This is illustrated by a systematic review by Sawada et al., showing that the initial diagnosis was mistaken as gastric cancer, submucosal tumor, GIST, or peptic ulcer disease in >50% of the patients, and resection occurred in 47.6% of the gastric IgG4-RD. CT abdomen showed a submucosal tumor and diffuse or focal wall thickening in most of the patients. Serum IgG4 levels were only available in half of the patients, with a median of 430 mg/dL (33).

For the diagnosis of IgG4-RD, a cutoff of serum IgG4 > 135 mg/dL is in generally widely accepted. A retrospective study of Masaki et al. in 132 patients showed a sensitivity and specificity of 97.0 and 79.6%, respectively, for this cutoff. Serum IgG4/IgG ratios above 8% have a sensitivity of 95.5% and a specificity of 87.5% for IgG4-RD (34).

The histopathological diagnosis of upper GI IgG4-RD is still a matter of debate. Uchino et al. used >10 IgG4-positive plasma cells per HPF and an IgG4/IgG-positive ratio > 40% as a cutoff for IgG4-high cases, based on 2020 revised comprehensive diagnostic criteria for IgG4-RD (29, 35). Importantly, there are organ-specific criteria for IgG4-RD, but these are not established for upper GI IgG4-RD. The ratio of IgG4/IgG-positive cells is more important for diagnosis than the absolute numbers because, in rheumatoid arthritis, atopic dermatitis and ANCA-associated vasculitis infiltration by IgG4-positive cells can be observed (29, 36).

A consensus statement on the pathology of IgG4-related disease by Deshpande et al. has put forward three main pathological features for the diagnosis of IgG4-related disease: storiform fibrosis, lymphoplasmacytic infiltration, and obliterative phlebitis. Plasma cells and lymphocytes are polyclonal (37). Eosinophils are common whereas neutrophilic infiltration, necrosis, and granuloma are atypical. Although only seen in 29–42% of the patients, bottom-heavy lymphoplasmacytic mucosal infiltration or bottom-heavy plasmacytosis (BPH) is characteristic for IgG4-gastritis (31, 35). In patients with known IgG4-RD, gastric biopsies of 31 patients were analyzed by Uchino et al. and were compatible with IgG4-high cases, i.e., both IgG4/IgG4 ratio > 40% and > 10 IgG4-positive plasma cells/HPF, in 10 cases (out of 25 eligible biopsies). In six cases, BHP was identified in the IgG4-high cases. Only one patient, under treatment with corticosteroids, out of the IgG4-low group showed BPH. It is noted that the evaluation of BHP can be difficult due to the disoriented sectioning (35). Uchino et al. (35) also found that permeation of plasma cells between non-atrophic fundic glands and plasmocytic aggregation in the muscularis mucosae is useful for the diagnosis of GI IgG4-related disease, as it was only rarely observed in the control cases. Moreover, striated inflammation in the muscularis propria is proposed as a characteristic feature of IgG4-related gastritis, as shown in surgically resected specimens (31).

#### Treatment of IgG4-related disease in general

No randomized clinical trials are available on the treatment of IgG4-RD, and certainly not in the field of upper GI Ig4-RD.

In general, corticosteroids are used as first-line treatment (38). A Japanese consensus statement paper for the treatment of autoimmune IgG4-related pancreatitis suggested a dose of 0.6 milligrams per kilogram (mg/kg) prednisolone per day for 2 to 4 weeks for the treatment of pancreatitis, followed by tapering over 3 to 6 months to a maintenance dose of 2.5–5 mg/day up to 3 years (39). Approximately 2 weeks after the start of corticosteroids, a follow-up serological assessment should be performed to objectify the decline in serum IgG4 (30).

Disease flare-ups are common despite treatment with glucocorticoids. Several publications have reported on the use of methotrexate, mycophenolate mofetil, and azathioprine as potential maintenance treatment options (38). In patients with refractory or recurrent disease, rituximab can be considered (38). Anti-TNF treatment has been described in two non-IBD patients with IgG4-RD with a good response (i.e., infliximab in IgG4-related orbital disease and adalimumab in IgG4-related colitis) (40, 41).

### Link between IgG4-RD and IBD

The role of IgG4 levels in patients with underlying IBD remains unclear. Published results are heterogenous when it comes to described associations between IgG4 and IBD, as shown in Table 1 (42–51). No research on upper GI IgG4-RD is available. In some patients with UC, IgG4 infiltration was associated with a worse outcome (44, 46). Whether IgG4 infiltration is a marker for aggressive IBD rather than a separate disease entity in these patients is still unknown. To date, a possible correlation is mainly suggested in patients with UC. Although little data are available to conclude.

**Table 1:** Studies reporting on associations between serum/mucosal IgG4 and IBD.

Author, year	Study design	Serum or mucosal IgG4 levels	IBD pts (UC/CD)	Control group	Findings
Koutroumpakis et al. (42)	Observational, monocentric study	Serum IgG4 (Ref 9–89 mg/dL)	N: 1193 (788 CD, 405 UC)	/	High serum IgG4 levels in 61/1193 (5%); no difference between CD and UC Association with PSC No correlation with disease extent or severity
Faria et al. (43)	Cross-sectional study	Serum IgG4 > 140 mg/dL; Colon tissue ≥10 IgG4+ plasma cells per field	N: 56 (26 CD, 30 UC)	/	High serum IgG4 levels: 9 UC, 1 CD (*p* = 0,006); no association with disease activity 3/26 increased number of colon tissue IgG4 plasma cells, no correlation with high IgG4 in serum
Wang et al. (44)	Case–control study	Serum IgG4 > 1.50 g/L; Mucosal >10 IgG4+ plasma cells/HPF	N: 232 (28 CD, 104 UC)	45 healthy controls	Serum IgG4: no significant difference between groups (*p* > 0.05), elevated in 9.9% of IBD pts. Higher mucosal IgG4 in UC and CD In UC: correlation between high mucosal IgG4 and serum IgG4 levels, disease extension and severity Mucosal IgG4 significantly decreased after treatment with glucocorticosteroids
Raina et al. (45)	Retrospective	Mucosal >10 IgG4+ plasma cells/HPF in rectal biopsies	N: 54 (13 CD, 18 UC, 23 UC + PSC)	11 controls (aspecific diarrhea or microscopic colitis)	Significantly elevated in active UC and UC + PSC (*p* = 0.05). Not in CD, inactive UC, and controls
Şimşek et al. (46)	Retrospective	Mucosal >10 IgG4+ plasma cells/HPF	N: 72 (17 CD, 55 UC)	/	Significantly higher mucosal IgG4 in UC than CD (*p* = 0.01), correlation with disease activity in UC
Keyashian et al. (47)	Retrospective	Mucosal IgG4+ plasma cells/HPF in rectal biopsies	N: 134 UC	/	Significant association between IgG4-positive plasma cells and histological disease activity (*p* = 0.001) No association between IgG4 counts and clinical outcome
Navaneethan et al. (48)	Retrospective	Serum IgG4 > 112 mg/dL	N: 50 PSC pts. of which 42 had UC	/	High IgG4 in 10 PSC pts. (20%); not significantly associated with UC (*p* = 0.067), significantly associated with younger age at PSC diagnosis, backwash ileitis, UC flares, and reduced colectomy-free survival
Tavaghi et al. (49)	Retrospective	Serum IgG4-levels	N: 73 PSC pts. of which 51 had IBD	/	No significant difference between IgG4 serum levels in PSC pts. with or without IBD
Navaneethan et al. (50)	Prospective	Serum IgG4 ≥ 112 mg/dL	N: 124 UC pts. with symptomatic pouchitis	/	8% (10/124) high serum IgG4 No significant association with auto-immune disorders or PSC Significantly more chronic antibiotic-refractory pouchitis in high serum IgG4 (*p* = 0.03)
Virk et al. (51)	Retrospective	Mucosal >10 IgG4+ plasma cells/HPF in pretreatment colonic biopsies	N: 78 (50 UC, 38 CD)	/	Significantly higher mucosal IgG4 in UC than CD (*p* = 0.0001) Significant correlation with histological activity, but not with disease extent.

IBD, inflammatory bowel disease; CD, Crohn’s disease, UC, ulcerative colitis, pts, patients; PSC, primary sclerosing cholangitis; HPF, high power field.

## Discussion

A thorough work-up remains necessary in patients presenting with refractory, ulcerative gastroduodenal inflammation, and differentiating between GG, upper GI IgG4-RD, and upper GI CD remains challenging. We report a case in which IgG4-RD gastroduodenitis and upper GI CD were overlapping. Granulomas are typical but not necessary in diagnosing CD after eliminating other causes such as sarcoidosis and tuberculosis. As was the case in our patient, GG-associated CD is more prevalent in male and young patients. On the other hand, granulomas are uncommon in IgG4-RD. However, due to persisting ulcerative gastroduodenitis with stenosis despite high doses of pantoprazole, an IgG4 stain and serum IgG4 were requested, which was positive. As shown in the literature, IgG4-RD should be ruled out not only in ulcerative disease but also in patients with a submucosal tumor or suspicion of GIST to avoid unnecessary surgery. First-line treatment for both CD and IgG4-RD consists of corticosteroids with tapering. Our patient received anti-TNF and azathioprine as a maintenance treatment based on the existing literature for CD, which is in favor of anti-TNF and/or azathioprine, and IgG4-RD, which favors an immunomodulator such as azathioprine. This approach led to an excellent response clinically, endoscopically, and histologically.

Physicians should be aware of the potential existence of IgG4-RD in the upper GI tract in refractory disease. Nonetheless, literature on diagnosis, prognosis, and treatment of upper GI IgG4-RD is scarce and the meaning of increased IgG4 in serum or gastric/duodenal biopsies remains the subject of further research.

Especially in IBD patients, mucosal IgG4-infiltration may be a marker and risk factor for aggressive disease rather than a separate disease entity, which is to date mostly suggested in UC. More research in this field is necessary to unravel this question and further explore the actual role of IgG4-elevation of IBD patients.

## Ethics statement

Written informed consent was obtained from the individual(s) for the publication of any potentially identifiable images or data included in this article.

## Author contributions

VD: Writing – original draft. JG: Writing – review & editing. AH: Writing – review & editing. TL: Writing – review & editing.
